# Information on food additives on food labels in Brazil: a critical analysis

**DOI:** 10.11606/s1518-8787.2023057004371

**Published:** 2023-02-08

**Authors:** Vanessa dos Santos Pereira Montera, Ana Paula Bortoletto Martins, Laís Amaral Mais, Daniela Silva Canella

**Affiliations:** I Universidade do Estado do Rio de Janeiro Instituto de Nutrição Programa de Pós-graduação em Alimentação, Nutrição e Saúde Rio de Janeiro RJ Brasil Universidade do Estado do Rio de Janeiro. Instituto de Nutrição. Programa de Pós-graduação em Alimentação, Nutrição e Saúde. Rio de Janeiro, RJ, Brasil; II Universidade de São Paulo Faculdade de Saúde Pública Núcleo de Pesquisas Epidemiológicas em Nutrição e Saúde São Paulo SP Brasil Universidade de São Paulo. Faculdade de Saúde Pública. Núcleo de Pesquisas Epidemiológicas em Nutrição e Saúde. São Paulo, SP, Brasil; III Instituto Brasileiro de Defesa do Consumidor São Paulo SP Brasil Instituto Brasileiro de Defesa do Consumidor. São Paulo, SP, Brasil; IV Universidade do Estado do Rio de Janeiro Instituto de Nutrição Departamento de Nutrição Aplicada Rio de Janeiro RJ Brasil Universidade do Estado do Rio de Janeiro. Instituto de Nutrição. Departamento de Nutrição Aplicada. Rio de Janeiro, RJ, Brasil

**Keywords:** Food Additives, adverse effects, Nutritional Facts, Food Labeling, legislation & jurisprudence

## Abstract

Questions about the safety of food additives and their consumption have been raised in recent years. The increased exposure to these substances, either by intake of ultra-processed foods or by the broad use and combination of various categories of additives, may be related to higher risks to consumer health. This article comments on the results of a study that quantified and characterized food additives found on the labels of 9,856 packaged foods and beverages available in Brazilian supermarkets. The study adopted a field diary method to record and analyze nonconformities in the lists of ingredients. The objective of this article is to discuss the use of additives identified on the labels and the limitations of Brazilian legislation, which should guarantee the right to information and health.

## INTRODUCTION

Food additive is any ingredient intentionally added to food during its manufacture, processing, packaging, storage, transport or handling, without the purpose of providing nutrition, instead modifying the food’s physical, chemical, biological or sensorial characteristics^1^. Additives are widely used in ultra-processed foods and beverages^2^ for various purposes, being considered as one of the markers of ultraprocessing^3^. Studies show that many foods and beverages packaged and available in Brazil, Australia and France contain food additives^4^.

The use of additives in Brazil is regulated by the National Health Surveillance Agency (Anvisa), which is based on other regional regulations, such as the Southern Common Market (Mercosur), and by committees of the World Health Organization (WHO) and the Food and Agriculture Organization (FAO) of the United Nations. Anvisa authorizes the use of several food additives^1^, but their effects on consumer health have been questioned^7^.

Questioning such effects is an important task since the consumption of these substances presents variation, as they are combined with other additives, either in the same item or in products consumed over the course of a day. This must be considered a critical issue in the recent scenario of growth in the intake of ultra-processed foods and beverages^18^.

A Brazilian study evaluated the distribution and pattern of food additives on the labels of 9,856 packaged foods and beverages in Brazilian supermarkets, concluding that 1/5 of the products did not contain any, whereas 1/4 had six or more food additives in their formulation. The existence of groupings of additives that were repeated in different food groups was verified, with emphasis on ultra-processed ones^4^. The quantification and characterization of food additives was made from labels^4^and a field diary method was used to record different information available in the ingredients list. The data were analyzed qualitatively, being compared to the current legislation in order to identify non-conformities. The information about these additives on labels was insufficient even when abiding to regulations. Lists of ingredients of ultra-processed foods (with food additives) were compared to the corresponding traditional culinary recipes—details of this step can be found in Montera^23^ (2021).

The objective of this article is to present a critical analysis of information about additives on food labels, aiming to foster an in-depth discussion about the use of these substances and the urgency to improve current legal standards.

### Presentation of Food Additives Found on Labels of Packaged Foods and Beverages Marketed in Brazil

Considering the different functions of food additives, flavoring substances is one of the categories in which most flaws were observed, considering their description and compliance with the current legislation^24^. According to the Brazilian norm, it is not necessary to declare the name of each substance that composes the flavoring or its mixture, and it is sufficient to designate them together with the word “flavoring” or “aroma,” identifying its classification as “natural,” “identical to natural” or “artificial.” However, several cases mentioned only “flavoring substances” in the ingredients list of products such as cereal bars, breakfast cereals and frozen meals. This nomenclature indicates a mixture of unidentified and unclassified additives. How to know if that is a mixture of natural or synthetic flavorings? This loophole allows the indiscriminate usage of food additives without consumer knowledge.

Another aspect regarding flavoring substances is that they may appear masked in the list of ingredients. In products such as ready-made sauces and seasonings, the expression “x-flavored prepared condiment,” being “x” some specific flavor, such as cooked meat or four cheeses, without any explanation about what such a “prepared condiment” is. In other products, after the “x-flavored prepared condiment,” an explanation of what the condiment is made of was seen—an example is in the list of ingredients of the “Brand A cream of vegetables (instant soup mixture)” (Chart). In that case, the detailing of the “ready-made condiment” noted the presence of flavoring substances and other food additives. In the “Brand B mashed potatoes and jerked beef mix,” the following information was present “ready-made parmesan flavored prepared condiment,” but no information about what the condiment is made of was available and the ingredients section did not list any parmesan cheese nor parmesan cheese flavoring. This leads us to question where that flavor comes from, since the sentence “ready-made parmesan flavored prepared condiment” may include the presence of other ingredients that confer such flavor.

**Chart:** List of selected foods and compound products.

Products	Ingredients
Brand A cream of vegetables (soup mixture)	Prepared vegetable-flavored condiment (salt, corn starch, corn syrup solids, condiment and herb, yeast extract, sodium glutamate flavor enhancer, anti-humectant silicon dioxide and tricalcium phosphate, acid regulator, citric acid and flavoring substances)
Brand E tomato-based sauce	Water, soybean oil, tomato extract, vinegar, sugar, modified starch, salt, mustard, whole egg, garlic, polysorbate stabilizer, nutmeg and paprika essential oil, mustard essential oil, prepared garlic condiment, sodium benzoate conservative, citric acid acidulant, sequestering agent EDTA and TBHQ antioxidant
Brand F pepper sauce with olive oil	Olive oil, refined vegetable oil, chilli pepper extract, garlic extract, paprika dye, aromas and tocopherol alpha antioxidant
Brand H frozen ham and champignon pizza	Wheat flour enriched with iron and folic acid, water, margarine, sugar, fresh organic yeast, refined salt, tomato paste, modified corn starch, garlic, soybean oil, parsley powder, mozzarella cheese, ham, champignon, black olive and oregano
Brand I cheese *empanada* with caramelized onions and requeijão cheese	Wheat flour enriched with iron and folic acid, maltodextrin, milk powder, dairy compound with vegetable fat milk flavor, corn starch, salt, garlic powder, nutmeg, titanium dioxide dye and monosodium glutamate flavor enhancer
Brand J ready-made mayonnaise	Water, vegetable oil, modified starch, vinegar, pasteurized egg, salt, lemon juice, lactic acid acidulant, preservative potassium sorbate, xanthan gum stabilizer, beta-carotene dye, sequestering agent EDTA, disodium calcium, TBHQ antioxidant and mustard aroma
*Baiano* seasoning	Coriander, black pepper, cumin, turmeric, pepperoni pepper, oregano and monosodium glutamate flavor enhancer
Curing salt	Sodium nitrite salt, color fixator and food preservative
Stabilizer	Stabilizers and thickeners

EDTA: ethylenediaminotetraacetate; TBHQ: terc-butyl-hydroquinone.

Other masked flavorings were identified. In the “Brand C oven baked rice with sausage and tomato sauce,” one item in the list of ingredients is “mozzarella-flavored starch.” In “Brand D instant noodles with Bolognese-flavored seasoning,” one finds “meat-flavored textured granulated soy protein.” In these cases, the ingredients list did not contain the foods providing the flavors—mozzarella and meat—and no flavoring substances were described. Starch has no mozzarella flavor, what would that represent in the ingredients list ? A food, a food additive, or both? In the two examples, flavoring substances are certainly present, but consumers are not made aware of which ones or their type(natural, identical to natural or synthetic).

According to the Resolution of the Collegiate Board (RDC) No. 2, of January 15, 2007^24^, flavoring substances are synonymous to aromatic compounds, essential oils and other agents (*saborizante* and *essência*, in Portuguese). Different technical terms to identify a flavoring substance means that information on this additive is unclear to consumers—as in the case of “Brand E tomato-based sauce” (Chart). The label is not clear in stating that the essential oils mentioned in the list are flavoring substances. Extracts can also be classified as flavoring substances, making their identification difficult—as seen in the “ Brand F pepper sauce with olive oil” (Chart). When reading the ingredients list, it is not evident that the product contains two flavoring substances, described as extracts, in addition to unspecified aromatic compounds.

The issue of extracts is even more complex since they can have more than one function. For instance: rosemary extract works as an antioxidant; yeast extract as a natural enhancer of salty flavor; malt extract, such as sugar syrup, provides color and taste; sage extract acts as a natural flavoring; meat extract as flavoring... But what kind? It is unclear what these extracts represent.

Another problem found was the inadequate description of flavoring substances in the ingredients list, going against the Brazilian legislation^24^. In the “Brand G chicken-flavored corn snack,” the ingredients list notes “aroma identical to natural chicken,” and, in parentheses, the explanation of what it is: “aromatic base, salt, monosodium glutamate flavor enhancer and soy derivative”. This does not represent an aroma identical to the natural. According to Anvisa^24^, this type of aroma actually refers to “chemically defined substances obtained by synthesis or isolated by chemical processes from raw animal, vegetable or microbial materials with a chemical structure identical to the substances in the natural raw materials—processed or not.”

Another important issue is the case of compound ingredients (when an ingredient is prepared from two or more other ingredients), for example, “*baiano* seasoning”—present in some foods, such as kibbeh, *coxinha*, salted croquettes—, “curing salt”—found in certain meats and fish—, and “stabilizer”—used in ice cream and chocolates—, the composition can be seen on the Chart. The current legislation requires a description, between parentheses, of each element of compound ingredients in the list of ingredients, with each individual described in descending order of proportion. However, when the name of a compound ingredient is present in a *Codex Alimentarius* standard or a specific technical regulation, and represents less than 25% of the food, it is not necessary to describe its individual ingredients, with the exception of additives of technological function in the final product. This practice, however, prevents consumers from recognizing the presence of food additives^25^.

Some foods do not contain food additives in the list of ingredients, but are composed of foods that typically contain them. For example, “Brand H frozen ham and champignon pizza” (Chart), may not seem to contain food additives, as they would not have been added directly to the food available for sale. However, many ingredients in the pizza are composed of various food additives, such as margarine, tomato paste, mozzarella cheese and ham. Costumers are thus exposed, without knowledge, to a combination of food additives. The situation is recurring in frozen meals or snacks—pizzas, pies, lasagna, pasta, sandwiches, croquettes, among others.

Finally, the differences between the composition of homemade culinary preparations and their non-homemade versions (ready-to-eat products) is worth noting. In a simple example, a homemade béchamel sauce traditionally uses milk, wheat flour, butter, nutmeg, salt and black pepper, whereas another version of the sauce found in “Brand I cheese *empanada* with caramelized onions and *requeijão* cheese,” as one of its ingredients (Chart). Another example is mayonnaise, which, in its homemade version contains whole egg, egg yolk, vegetable oil, vinegar, mustard and salt, quite different from the composition of “Brand J ready-made mayonnaise” (Chart).

The claim that ultra-processed foods are simply the result of processing that allows their consumption, similar to what occurs in the domestic environment^26^, is unreliable. In fact, ultra-processed foods and those submitted to domestic processing differ not only by the extent and purpose of the processing, or by the number of ingredients, but also by the types of ingredients that compose them. Food additives found in ultra-processed foods and beverages are not used in homemade preparations. For example, homemade béchamel sauce does not need to be made whiter or have its flavor made stronger with a flavor enhancer.

## FINAL CONSIDERATIONS

The information about food additives on the labels of packaged foods and beverages marketed in Brazil is not sufficiently clear, and is often inadequate, to the point of disrespecting the consumer’s right to know the composition of foods and the risks they may present to their health. This prevents the freedom to make conscious choices at the time of purchase. Moreover, the Consumer Protection Code establishes, in Article 6, item III, that “adequate and clear information about the different products and services, with correct specification of quantity, characteristics, composition, quality, taxes and price, as well as the risks they present” must exist in the labels^27^. The objective is to ensure consumer safety and prevent suppliers—in this case, the industry of ultra-processed foods—from using strategies to inscribe their product with misleading information. Thus, it is necessary to rethink the Brazilian legislation on food additives to improve the rules of general food labeling.
