# Supplementary material for: Information on food additives on food labels in Brazil: a critical analysis
Source: Rev Saude Publica. 2023 Feb 8;57:2. doi: 10.11606/s1518-8787.2023057004371 (PMC9933643; doi:10.11606/s1518-8787.2023057004371)
Supplement: Translation

# Translation: Informação sobre aditivos alimentares nos rótulos de alimentos no Brasil: análise crítica

**Authors:** Vanessa dos Santos Pereira Montera, Ana Paula Bortoletto Martins, Laís Amaral Mais, Daniela Silva Canella

**Affiliations:** I Brasil Universidade do Estado do Rio de Janeiro. Instituto de Nutrição. Programa de Pós-graduação em Alimentação, Nutrição e Saúde. Rio de Janeiro, RJ, Brasil; II Brasil Universidade de São Paulo. Faculdade de Saúde Pública. Núcleo de Pesquisas Epidemiológicas em Nutrição e Saúde. São Paulo, SP, Brasil; III Brasil Instituto Brasileiro de Defesa do Consumidor. São Paulo, SP, Brasil; IV Brasil Universidade do Estado do Rio de Janeiro. Instituto de Nutrição. Departamento de Nutrição Aplicada. Rio de Janeiro, RJ, Brasil

**Keywords:** Aditivos Alimentares, efeitos adversos, Informação Nutricional, Rotulagem de Alimentos, legislação & jurisprudência

## Abstract

Nos últimos tempos, questões vêm sendo levantadas sobre a segurança no uso de aditivos alimentares e em seu consumo. verificou-se que o aumento da exposição a essas substâncias, seja pela ingestão mais frequente de alimentos ultraprocessados ou pela ampla utilização e combinação de várias categorias de aditivos pela indústria, pode estar relacionado ao maior risco à saúde do consumidor. Um estudo quantificou e caracterizou aditivos alimentares encontrados nos rótulos de 9.856 alimentos e bebidas embalados disponíveis nos supermercados brasileiros. nele, foi adotado um diário de campo para registro de falhas e inconformidades nas informações presentes nas listas de ingredientes, sendo, por fim, analisadas qualitativamente e descritas de forma narrativa. Com base nisso, o objetivo deste comentário é apresentar e discutir o uso desses aditivos identificados nos rótulos e as limitações da legislação brasileira, que deveria garantir o direito à informação e a saúde da população.

## INTRODUÇÃO

Aditivo alimentar é todo e qualquer ingrediente adicionado intencionalmente aos alimentos durante sua fabricação, processamento, preparação, tratamento, embalagem, acondicionamento, armazenagem, transporte ou manipulação, sem o propósito de nutrir, mas com o objetivo de modificar as suas características físicas, químicas, biológicas ou sensoriais^1^. Os aditivos são amplamente utilizados em alimentos e bebidas ultraprocessados^2^ para diversas finalidades; são, inclusive, considerados como um dos marcadores do ultraprocessamento^3^. Aliás, estudos já avaliaram que uma grande quantidade de alimentos e bebidas desse tipo, embalados e disponíveis para o consumo no Brasil, Austrália e França, possuem aditivos alimentares^4^.

No Brasil, o uso desses aditivos é regulado pela Agência Nacional de Vigilância Sanitária (Anvisa), que se fundamenta também em critérios apoiados por regulamentações regionais, como as do Mercado Comum do Sul (Mercosul), e em sugestões emitidas a nível mundial por comitês de especialistas da Organização Mundial de Saúde e da Organização das Nações Unidas para Alimentação e Agricultura^1^. Entretanto, apesar de diversos aditivos alimentares serem autorizados pela Anvisa^1^, seus efeitos sobre a saúde do consumidor vêm sendo questionados^7^.

Questionar esses efeitos torna-se ainda mais importante quando se nota uma variação na condição de consumo dessas substâncias, pois, na prática, elas não são consumidas isoladamente, mas combinadas a outros aditivos, seja em um mesmo alimento ou em alimentos consumidos ao longo de um dia. Sendo assim, esse problema deve ser considerado crítico em um cenário de crescimento da ingestão de alimentos e bebidas ultraprocessados^18^.

Nesse contexto, um estudo brasileiro avaliou a distribuição e o padrão de uso dos aditivos alimentares em rótulos de 9.856 alimentos e bebidas embalados disponíveis nos supermercados brasileiros, concluindo que 1/5 dos produtos não continham qualquer aditivo alimentar, enquanto 1/4 tinha seis ou mais em sua formulação. Além disso, verificou-se a existência de agrupamentos de aditivos que se repetiam em diferentes grupos de alimentos, com destaque, claro, para os ultraprocessados^4^. Nesse estudo, foi feita a quantificação e caracterização dos aditivos alimentares a partir dos rótulos^4^ e, complementando a análise, foi adotado um diário de campo que registrou diferentes informações disponíveis na lista de ingredientes. Os dados oriundos do registro foram analisados qualitativamente e descritos de forma narrativa, sendo comparados à legislação vigente, referente ao uso de aditivos alimentares, a fim de identificar falhas e inconformidades com as normas específicas. A partir disso, constatou-se a insuficiência de informação sobre esses aditivos nos rótulos, mesmo em situações de regularidade. Enfim, as listas de ingredientes de alimentos ultraprocessados, em que tinham aditivos, foram comparadas com as receitas culinárias tradicionais correspondentes – detalhes dessa etapa podem ser encontrados em Montera^23^ (2021).

Considerando o desenvolvido até aqui, o objetivo deste comentário é apresentar uma análise crítica acerca das informações sobre aditivos alimentares em rótulos de alimentos, visando fomentar uma discussão aprofundada em torno do uso dessas substâncias e da urgência em se aprimorar as normas legais vigentes.

### Apresentação dos Aditivos Alimentares Encontrados nos Rótulos de Alimentos e Bebidas Embalados Comercializados no Brasil

Considerando as diferentes funções dos aditivos alimentares, os aromatizantes representam uma das categorias nas quais mais se observaram pontos falhos, tanto na sua descrição quanto na sua conformidade com a legislação vigente^24^. Segundo a norma brasileira, não é necessário declarar o nome de cada substância que compõe o aroma ou a mistura de aromas, sendo suficiente designá-las em conjunto com a palavra “aromatizante” ou “aroma”, identificando sua classificação como “natural”, “idêntico ao natural” ou “artificial”. Contudo, em diversos casos, encontrou-se apenas a menção a “aromatizantes” ou “aromas” na lista de ingredientes de produtos, como barras de cereais, cereais matinais e refeições prontas. Isso é um indicativo que há nesses produtos uma mistura de aditivos não identificados nem tipificados. Como, então, saber se aquela é uma mistura de aromatizantes naturais ou sintéticos? Surge, aqui, uma brecha que permite a utilização indiscriminada de aditivos alimentares e a ausência de uma comunicação adequada para ciência do consumidor.

Um outro aspecto percebido em relação aos aromatizantes é que, em alguns casos, eles podem aparecer de forma mascarada na lista de ingredientes. Em produtos como molhos e temperos prontos encontramos a expressão “condimento preparado sabor x”, sendo “x” algum sabor específico, como carne cozida ou quatro queijos, sem qualquer explicação sobre o que é o tal “condimento preparado”. Em outros produtos, encontrou-se, após o item “condimento preparado sabor x”, parênteses explicando do que se trata o condimento – isso pode ser visto, por exemplo, na lista de ingredientes do “creme de legumes (mistura para preparo de sopa) marca A” (Quadro). Nota-se que, nesse caso, houve um detalhamento do que era o “condimento preparado” e que, nas misturas que formam tais condimentos, há a presença de aromatizantes, dentre outros aditivos alimentares. Já na “mistura para o preparo de purê de batata com charque marca B”, encontrou-se “condimento preparado sabor parmesão”, porém, não somente não havia o detalhamento sobre o que é o “condimento preparado” em questão, como não constava na lista de ingredientes qualquer queijo parmesão ou aromatizante sabor parmesão. Isso nos leva a questionar de onde vem esse sabor. Baseado na observação da lista de ingredientes de outros alimentos, provavelmente, por trás da expressão “condimento preparado sabor parmesão” exista um aromatizante não identificado, assim como outros possíveis ingredientes e aditivos também não identificados que confiram tal sabor ao alimento tratado.

**Quadro:** Lista de ingredientes de alimentos e produtos compostos selecionados.

Produtos	Ingredientes
Creme de legumes (mistura para preparo de sopa) marca A	Condimento preparado sabor vegetais (sal, amido de milho, sólidos de xarope de milho, condimento e erva, extrato de levedura, realçador de sabor glutamato de sódio, antiumectante dióxido de silício e fosfato tricálcico, regulador de acidez ácido cítrico e aromatizantes)
Molho à base de tomate marca E	Água, óleo de soja, extrato de tomate, vinagre, açúcar, amido modificado, sal, mostarda, ovo integral, alho, estabilizante polisorbato, óleo essencial de noz moscada e páprica, óleo essencial de mostarda, condimento preparado de alho, conservador benzoato de sódio, acidulante ácido cítrico, sequestrante EDTA e antioxidante TBHQ
Molho de pimenta com azeite marca F	Azeite de oliva, óleo vegetal refinado, extrato de pimenta malagueta, extrato de alho, corante páprica, aromas e antioxidante alfa tocoferol
Pizza de presunto com champignon congelada marca H	Farinha de trigo enriquecida com ferro e ácido fólico, água, margarina, açúcar, fermento biológico fresco, sal refinado, polpa de tomate, amido de milho modificado, alho, óleo de soja, salsa em pó, queijo muçarela, presunto, champignon, azeitona preta e orégano
Empanada de queijo com cebola caramelada com catupiry marca I	Farinha de trigo enriquecida com ferro e ácido fólico, maltodextrina, leite em pó, composto lácteo com gordura vegetal sabor leite, amido de milho, sal, alho em pó, noz moscada, corante dióxido de titânio e realçador de sabor glutamato monossódico
Maionese pronta da marca J	Água, óleo vegetal, amido modificado, vinagre, ovo pasteurizado, sal, suco de limão, acidulante ácido lático, conservador sorbato de potássio, estabilizante goma xantana, corante beta-caroteno, sequestrante EDTA cálcio dissódico, antioxidante TBHQ e aroma de mostarda
Tempero baiano	Coentro, pimenta preta, cominho, cúrcuma, pimenta calabresa, orégano e realçador de sabor glutamato monossódico
Sal de cura	Sal com nitrito de sódio, um fixador de cor e conservante
Liga neutra	Estabilizantes e espessantes

EDTA: etilenodiaminotetraacetato; TBHQ: terc-butil-hidroquinona.

Outros aromatizantes mascarados foram identificados. No “arroz de forno com calabresa e molho de tomate marca C”, há na lista de ingredientes “amido sabor muçarela”. Em outro produto, “macarrão instantâneo com tempero sabor bolonhesa marca D”, encontra-se a “proteína texturizada granulada de soja sabor carne”. Nesses casos, a lista de ingredientes não continha os alimentos conferindo os sabores indicados – muçarela e carne – e não havia aromatizantes descritos. Visto que amido não tem sabor de muçarela, o que isso representaria na lista de ingredientes? Um alimento, um aditivo alimentar, ou os dois? Em ambos os exemplos, certamente existem aromatizantes, mas não estão explicitados para o consumidor, muito menos é informado qual o tipo desse aromatizante – se natural, idêntico ao natural ou sintético.

Ainda, também de acordo com a Resolução da Diretoria Colegiada (RDC) nº 2, de 15 de janeiro de 2007^24^, consideram-se sinônimos de aromatizantes: aroma, óleo essencial, saborizante e essência. A utilização de diferentes termos técnicos para identificar um aromatizante faz com que a informação sobre a presença desse aditivo não seja clara para o consumidor – como no caso do “molho à base de tomate marca E” (Quadro). Não é óbvio para quem consome o produto que os óleos essenciais citados na lista são aromatizantes. Da mesma forma, extratos também podem ser classificados como aromatizantes, não facilitando a sua identificação – como visto no “molho de pimenta com azeite marca F” (Quadro). Ao ler a lista de ingredientes, não fica evidente que naquele produto existem dois aromatizantes, descritos como extratos, além dos aromas não especificados.

A questão dos extratos é ainda mais complexa, visto que podem ter mais de uma função. Por exemplo: extrato de alecrim funciona como antioxidante; extrato de levedura, como realçador natural do sabor salgado; extrato de malte, como xarope de açúcar que confere cor e sabor aos alimentos; extrato de sálvia atua como aromatizante natural; extrato de carne age como aromatizante… Mas de que tipo? Mais uma vez, não é claro o que representam esses extratos, principalmente para quem vai adquirir e consumir o alimento.

Um outro problema percebido na análise da lista de ingredientes foi a inadequação da descrição de aromatizantes, em relação à legislação brasileira^24^. No “salgadinho de milho sabor frango marca G”, encontrou-se na lista de ingredientes “aroma idêntico ao natural de frango”, e, entre parênteses, a explicação do que consiste esse aroma: “base aromática, sal, realçador de sabor glutamato monossódico e derivado de soja”. Isso não representa um aroma idêntico ao natural. De acordo com a definição da Anvisa^24^, esse tipo de aroma se refere, na verdade, a “substâncias quimicamente definidas obtidas por síntese e aquelas isoladas por processos químicos a partir de matérias primas de origem animal, vegetal ou microbiana, que apresentam uma estrutura química idêntica às substâncias presentes nas referidas matérias-primas naturais – processadas ou não”.

Outra questão importante a ser analisada é o caso dos ingredientes compostos, ou seja, quando um ingrediente é elaborado a partir de outros dois ou mais ingredientes, por exemplo, o “tempero baiano” – presente em alguns alimentos, como quibe, coxinha, bolinhos salgados –, o “sal de cura” – encontrado em determinadas carnes e peixes –, e a “liga neutra” – comumente usada em sorvetes e chocolates, cuja composição pode ser vista no Quadro. A legislação vigente determina que os ingredientes compostos sejam declarados na lista de ingredientes acompanhados imediatamente de uma outra listagem, entre parênteses, na qual os ingredientes individuais sejam descritos em ordem decrescente de proporção. Contudo, quando um ingrediente composto tem um nome estabelecido em uma norma do *Codex Alimentarius* ou em um regulamento técnico específico, e representar menos que 25% do alimento, não é necessário declarar seus ingredientes individuais, com exceção dos aditivos que desempenhem uma função tecnológica no produto final. Porém, essa prática acaba por impedir o consumidor de reconhecer a presença de aditivos alimentares^25^.

Vale, também, pontuarmos que alguns alimentos não contêm aditivos alimentares na lista de ingredientes, mas são compostos por alimentos que tipicamente os contém. Um exemplo é a “pizza de presunto com champignon congelada marca H” (Quadro). Esse produto aparenta não conter aditivos alimentares, pois eles não teriam sido adicionados diretamente ao alimento disponível para venda. No entanto, vários dos ingredientes que compõem a pizza são compostos por diversos aditivos alimentares, como a margarina, a polpa de tomate, o queijo muçarela e o presunto. Então, ao se consumir esse produto, o indivíduo está exposto a uma combinação de aditivos alimentares sem saber. E essa situação ocorre com frequência: em refeições ou lanches prontos… – pizzas, quiches, lasanhas, massas recheadas, escondidinhos, sanduíches, salgados, entre outros.

Por fim, cabe destacar diferenças entre a composição de preparações culinárias caseiras e suas versões não caseiras, que são parte de produtos prontos para o consumo. Um exemplo simples: quando se prepara um molho bechamel em casa, tradicionalmente utiliza-se leite, farinha de trigo, manteiga, noz moscada, sal e pimenta-do-reino – outra versão do molho pode ser vista na “empanada de queijo com cebola caramelada com catupiry marca I”, como um de seus ingredientes (Quadro). Outro exemplo é o da maionese. Uma maionese caseira contém ovo inteiro, gema de ovo, óleo vegetal, vinagre, mostarda e sal, bastante diferente da composição da “maionese pronta da marca J” (Quadro).

Nota-se que a alegação de que alimentos ultraprocessados são simplesmente resultado de processamentos que permitem seu consumo, similar ao que ocorre no meio doméstico^26^, não parece fidedigna. Na prática, alimentos ultraprocessados e aqueles submetidos ao processamento doméstico diferem não apenas pela extensão e propósito do processamento, ou pelo número de ingredientes, mas também pelos tipos de ingredientes que os compõem. Em preparações caseiras, não se utiliza os aditivos alimentares encontrados na lista de ingredientes de alimentos e bebidas ultraprocessados. Não é necessário deixar, por exemplo, um molho branco caseiro mais branco ou ressaltar seu sabor com um realçador de sabor para torná-lo mais atrativo.

## CONSIDERAÇÕES FINAIS

A informação a respeito dos aditivos alimentares nos rótulos de alimentos e bebidas embalados comercializados no Brasil não é clara o suficiente, e muitas vezes é inadequada, ao ponto de representar um desrespeito ao direito do consumidor de conhecer a composição dos alimentos disponíveis no mercado e os riscos que eles possam apresentar à sua própria saúde. Isso impede a liberdade de fazer escolhas conscientes no momento da compra. Aliás, vale ressaltar que o Código de Defesa do Consumidor estabelece no artigo 6º, inciso III, que haja “a informação adequada e clara sobre os diferentes produtos e serviços, com especificação correta de quantidade, características, composição, qualidade, tributos incidentes e preço, bem como sobre os riscos que apresentem”^27^. Com isso, busca-se garantir a segurança do consumidor e impedir que o fornecedor – nesse caso, a indústria de ultraprocessados – se utilize de estratégias para qualificar seu produto com informações enganosas. Assim, é preciso repensar a legislação brasileira referente a aditivos alimentares para que as regras de rotulagem geral de alimentos referente à lista de ingredientes sejam melhoradas.
